# The Significance of Palliative Care in Managing Pain for Patients Undergoing Hemodialysis

**DOI:** 10.3390/jcm14207129

**Published:** 2025-10-10

**Authors:** Nóra Szigeti, Botond Csiky, Ágnes Csikós, Balázs Sági

**Affiliations:** 1 2nd Department of Medicine and Nephrology-Diabetes Center, Medical School, University of Pécs, 7624 Pécs, Hungary; szigeti.nora@pte.hu (N.S.); csiky.botond@pte.hu (B.C.); sagi.balazs@pte.hu (B.S.); 2 National Dialysis Center Pécs, 7624 Pécs, Hungary; 3 Institute of Primary Health Care, Department of Palliative Medicine, Medical School, University of Pécs, 7623 Pécs, Hungary

**Keywords:** end-stage kidney disease, hemodialysis, pain, palliative care

## Abstract

**Background/Objectives:** Pain is a common issue among patients undergoing hemodialysis (HD), and palliative care (PC) aims to improve their quality of life. This study investigates the incidence, nature, and treatment of pain in chronic HD patients in Hungary, along with factors influencing pain intensity and the benefits of PC. **Methods:** This study used a cross-sectional design involving 159 patients with chronic end-stage kidney disease (ESKD) receiving HD at the National Dialysis Center in Pécs, Hungary. Pain was assessed using a “PQRST” questionnaire, and statistical analyses were performed on clinical and laboratory data to identify potential pain triggers. We also reviewed the latest literature on PC for patients with ESKD undergoing HD. **Results:** Approximately 57% of patients reported pain, with 88% indicating moderate-to-severe pain levels. While 64% used regular pain medication, only 29% experienced complete pain relief. Non-pharmacological methods, along with adjuvant agents and strong notably different based on parathyroid opioids, were underutilized. Pain intensity was hormone (PTH) and C-reactive protein (CRP) levels. Key factors affecting pain included body mass index (BMI), hypertension (HT), diabetes mellitus (DM), and PTH levels. Research shows that PC is rarely used for patients on HD in many countries, despite being effective in managing symptoms. **Conclusions:** PC, along with pain assessment and multidisciplinary management, reduces the symptomatic burden for patients with ESKD. Effective management of mild pain should be handled by a nephrologist experienced in PC, while severe, therapy-resistant pain should be managed by PC specialists. Therefore, implementing PC is essential in the treatment of these patients.

## 1. Introduction

Chronic kidney disease (CKD) currently affects more than 10% of the global population, and its prevalence is continuously rising. Several chronic illnesses and conditions can result in end-stage kidney disease (ESKD). Hemodialysis (HD) is the most widespread renal replacement therapy for this condition, but as kidney function declines, patients often experience numerous complications, resulting in burdensome symptoms [1]. Patients with progressive, chronic, and non-malignant illnesses, such as ESKD, experience a similar symptom burden to those with advanced cancer who are receiving palliative care (PC) [2,3].

Kidney supportive care (KSC) must be applied to all individuals with advanced kidney disease. Similar to PC in oncology, its primary goal is to reduce suffering throughout the disease, as well as at the end of life with hospice care. KSC is most effectively delivered through a collaborative approach that involves nephrologists applying “primary PC” skills for the routine assessment and management of symptoms. For complex cases that present a significant and challenging symptom burden, such as severe pain, consultation with PC specialists is recommended. Randomized prospective trials have demonstrated significant improvements in symptom burden, quality of life (QoL), functional status, and reductions in depression and anxiety for individuals receiving PC compared to those who only receive standard specialty care [3,4,5,6]. PC programs are most effective in Canada, the United Kingdom, Australia, New Zealand, and Hong Kong [3]. However, surveys conducted in Australia, New Zealand, and the United Kingdom indicate that more than a third of nephrology units lack dedicated KSC services [7]. In Australia, 72% of HD patients who could have potentially benefited from PC do not receive it [8], and in Hungary, as in several other countries [9,10,11,12,13,14], a specialized PC for patients with ESKD is not available.

Research indicates that 50% of patients with ESKD undergoing HD experience pain [2,3,15], but information regarding its origin, characteristics, and management remains relatively scarce [16,17,18,19,20,21,22].

In December 2023, we conducted a study to evaluate the symptom burden of 168 patients undergoing chronic HD. The first part of our study evaluated the changes in symptom burden among chronic HD patients with cancer using the Edmonton Symptom Assessment System Revised Renal (ESAS-r: Renal) questionnaire. However, the use of the survey was discontinued because the patients found it challenging to assess their symptoms using the questionnaire’s broad (0–10) scale. Consequently, in the second part of our study, we assessed the symptom burden of chronic HD patients, many of whom have poor cognitive function, using the 0–4 scale of the Integrated Palliative Care Outcome Scale Renal (IPOS-Renal) questionnaire. The average age of the participants was 65 (±12) years, and the average duration of dialysis treatment was 64 (±55) months. The underlying diseases that caused ESKD included diabetes (DM) (24%), polycystic kidney disease (22%), hypertension (HT) (20%), glomerulonephritis (20%), and other or unknown causes (14%). The Charlson Comorbidity Index, which assesses severe comorbid conditions, had a mean score of 6 (±2), indicating a high level of comorbidity and a 98% ten-year mortality rate. Additionally, responses to the “surprise question” indicated a 27% one-year mortality rate. In this patient population, the most significant physical symptom reported was pain, with 52% of patients indicating this issue [9].

Based on these findings from the IPOS-Renal study conducted in 2023, we conducted a cross-sectional study to explore the incidence and characteristics of pain and the pharmacological treatments utilized by our HD patients. To improve our understanding of the potential factors that trigger pain, we compared clinical data and routine laboratory parameters with the intensity of pain experienced. We aimed to evaluate the potential benefits of providing specialized PC for patients undergoing HD in our country by studying the recent literature on PC for ESKD patients [10,11,12,13,14,23,24].

## 2. Materials and Methods

### 2.1. Study Design

We conducted a cross-sectional, single-center study for patients older than 18 years of age who were undergoing chronic HD treatment—defined as a dialysis program for at least 3 months—at the National Dialysis Center of Pécs. Patients who were unable to complete the questionnaire due to their mental state were excluded from the study. This study was conducted in accordance with the Declaration of Helsinki and approved by the Institutional Ethics Committee of the Medical School of Pécs (Reference No. 8825-PTE2021, on 11 June 2021), and written informed consent was obtained from all patients involved in the study.

### 2.2. Patient Population

In 2024, 159 patients older than 18 years of age undergoing chronic HD were included in our study.

### 2.3. Pain Assessment

To evaluate patients’ pain experiences, we employed the “PQRST” approach for symptom assessment. “PQRST” stands for Provokes and Palliates, Quality, Region and Radiation, Severity, and Time [25]. The acronym “PQRST” represents the following elements of pain assessment:

- Provokes: What makes the pain worse?
- Palliates: What helps relieve the pain?
- Quality: What is the nature of the pain?
- Region: Where is the most intense pain located?
- Radiation: Where does the pain radiate?
- Severity: How intense is the pain? This is typically measured using the Numeric Rating Scale (NRS).
- Time: When does the pain occur?

It is also important to consider the negative impact of pain on a person’s life.

The PQRST approach for pain evaluation is a useful clinical tool, but it is not a validated research instrument like the Brief Pain Inventory (BPI), which would allow greater comparability with other studies. We selected this assessment method because, in our previous study conducted in 2023, patients found the ESAS-r: Renal questionnaire, which uses a scale of 0–10 like the BPI, to be too difficult to complete [9].

### 2.4. Factors Triggering Pain

To better understand the possible factors triggering pain, we compared clinical data and routine laboratory parameters with the intensity of the pain experienced. Clinical data indicated the presence of DM and HT, along with information on body mass index (BMI). Laboratory parameters included laboratory tests such as sodium, potassium, urea nitrogen, creatinine, calcium, phosphorus, parathyroid hormone (PTH), blood count, C-reactive protein (CRP), liver function tests, total protein, albumin, and lipid parameters. All of these tests were performed every three months during patient monitoring, and we compared our results with literature data on patients with ESKD who received PC [8,13].

### 2.5. Statistical Analysis

Statistical analyses were conducted using SPSS software version 21.0 (SPSS Inc., Chicago, IL, USA, 2020). The data are presented as means with standard deviations (SDs) and as percentages. We utilized Student’s *t*-test and ANOVAs, as needed, to compare clinical and laboratory parameters. The average SD represented information from a Gaussian distribution. The factors that influence the pain intensity were investigated using univariate and multivariate linear regression analysis. Values of *p* < 0.05 were considered statistically significant.

## 3. Results

Table 1 summarizes the baseline clinical data and pain presence in 159 patients with ESKD on HD.

The mean age of the patients was 65 (±12) years, with 80 (50%) being men. A total of 91 patients (57%) reported experiencing pain.

We conducted a detailed examination of the pain characteristics in these 91 patients. In 36 cases (40%), patients reported pain in several different regions. The characteristics of the pain varied across these areas, which explains why the total percentage of different characteristics can exceed 100%.

Table 2 summarizes the severity of pain in various areas, categorized by the NRS.

The results show that 15 patients (16%) experienced mild pain (NRS 1–3), 39 patients (43%) had moderate pain (NRS 4–6), and 41 patients (45%) reported severe pain (NRS 7–10).

**Figure 1:**
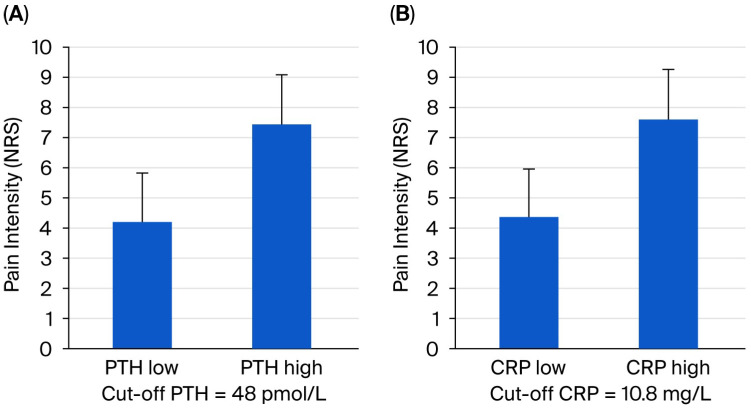
Pain intensity (NRS) in different groups of patients divided by PTH (**A**) and CRP (**B**) medians. NRS: Numeric Rating Scale; PTH: parathyroid hormone; CRP: C-reactive protein.

To enhance our understanding of the factors that trigger pain, we compared clinical data and routine laboratory parameters with the intensity of pain experienced. Patients were divided into two groups based on median PTH and CRP, showing a significant difference in pain intensity between individuals with low and high values (cut-off values: PTH = 48 pmol/L and CRP = 10.8 mg/L; *p* < 0.01) (Figure 1).

The significant confounding factors of intensity of pain included BMI (OR = 2.296; 95% CI: 1.019–2.981, *p* = 0.012), HT (OR: 7.93; 95% CI: 1.207–14.658, *p* < 0.05), DM (OR = 7.375; 95% CI: 1.917–13.833, *p* = 0.013), and PTH (OR = 1.578; 95% CI: 1.089–2.236, *p* = 0.027) (Table 3).

The details regarding the location and characteristics of the pain are summarized in Table 4.

The most common sites of pain were the hip and lower limb, affecting 50 patients (55%), followed by the back, reported by 20 patients (22%), and the shoulder and arm, also reported by 20 patients (22%). Pain localized to the head and neck was experienced by 18 patients (20%), while 16 patients (18%) reported distal foot pain, 15 patients (16%) reported waist pain, and 10 patients (11%) experienced pain in the gluteal region. Pain in the abdominal area was reported by nine patients (10%), distal hand pain by seven patients (8%), and chest pain by six patients (7%).

In terms of pain characteristics, 28 patients (31%) reported experiencing sharp pain, while 22 patients (24%) reported cramping pain, and another 22 (24%) described aching pain. Additionally, 16 patients (18%) experienced dull pain, 15 patients (16%) reported numbness, 12 patients (13%) experienced stabbing pain, 8 patients (9%) described throbbing pain, and 6 patients (7%) reported burning sensations.

Furthermore, 38 patients (42%) noted that their pain was constant, and 27 patients (30%) described their pain as radiating.

Although our primary objective was not to categorize the pain types, the nature of the reports indicated that nociceptive pain (sharp, cramping, aching, dull, and throbbing) was the most prevalent. However, neuropathic pain (numbness, stabbing, and burning) also occurred frequently, affecting 36% of the patients.

Seventy-three patients (80%) suffered from chronic pain lasting more than three months, while sixty-three patients (69%) experienced pain for over a year. Twelve men and twenty-one women reported experiencing severe pain for more than a year.

Factors that influence pain and its impact on quality of life are summarized in Table 5.

Pain increased with movement in 46 cases (51%), while it intensified with rest in 12 patients (13%). Three patients (3%) noted a connection between their pain and HD treatment, three patients (3%) linked it to weather changes, and two patients (2%) associated it with food. Rest reduced pain for 33 patients (36%), and movement alleviated it in 16 patients (18%).

Specifically, 57 patients (63%) reported difficulties with physical activity, and 44 patients (48%) had disturbed sleep, worsening their nutritional, physical, and psychological conditions. Fourteen patients (15%) reported loss of appetite, 13 patients (14%) faced emotional challenges, 10 patients (11%) mentioned difficulties with attention, and 5 patients (5%) experienced relationship issues.

The medications used for pain management and their effectiveness are listed in Table 6.

A total of 58 patients, representing 64% of those experiencing pain, used regular medication to manage their pain, sometimes in combination. However, none reported using non-pharmacological methods. Among these 58 patients, 31 (53% of those using medication) were taking non-steroidal anti-inflammatory drugs (NSAIDs), 22 (38%) were using metamizole, 3 patients (5%) were on paracetamol, and 13 (22%) were using weak opioids such as tramadol. No patients used strong opiates or adjuvant medications.

Of the patients receiving treatment, pain was successfully eliminated in 17 patients, representing 29%.

## 4. Discussion

Pain is a common symptom among patients with ESKD. Research shows that 50% of patients undergoing HD experience pain, with over 80% of those reporting moderate-to-severe pain [2,3,15,26]. In comparison, the prevalence of pain in patients with advanced metastatic cancer is 55%, but these patients may receive PC [2,3,27].

Our study indicated that 57% of patients reported experiencing pain, and among those, 88% indicated that the severity of their pain was moderate to severe, aligning with previously published data [2,3,15,26].

Some studies have examined the relationship between pain intensity and both clinical data and laboratory results in patients undergoing HD. Several factors have been associated with the experience of pain in these patients, including older age [19], female gender, high comorbidity indices, numerous painful sites, dialysis duration exceeding 24 months [17], increased BMIs [21], and the presence of diabetic retinopathy and neuropathy, as well as elevated levels of intact PTH [16,22], calcium [16], and CRP [20], and decreased levels of calcitriol [16], hemoglobin [18], and serum albumin concentration [20].

Our study found a significant difference in pain intensity between individuals with low and high levels of PTH and CRP. We also observed a strong correlation between pain intensity and factors such as DM, HT, BMI, and PTH levels.

DM and HT can cause pain through various mechanisms, including nerve damage (neuropathy) and reduced blood flow (ischemic changes) [28]. Higher BMIs may worsen chronic pain by lowering the pain threshold and increasing sensitivity, and various endocrine changes associated with obesity can affect pain modulation [21]. CRP serves as an objective measure of inflammatory activity in ESKD, accurately reflecting the production of pro-inflammatory cytokines [20]. Moreover, increased PTH levels may lead to skeletal pain due to renal osteodystrophy [22]. Overall, DM, HT, and higher BMIs significantly contribute to the development of common neuropathic pain [21,28].

The most common source of pain among HD patients is musculoskeletal issues [26], which are often linked to a high incidence of bone disease, bone fragility, and the progressive loss of muscle mass [29]. A survey conducted by Fleishman and colleagues identified the most common locations of pain as the lower limbs and the lower back. Headaches are a common issue for HD patients [17], and abdominal pain is prevalent, though its cause remains unclear [29].

Our study, consistent with the existing literature, found that musculoskeletal pain was the most common issue, particularly in the hip and lower limbs. Headaches and abdominal pain were also frequently reported among the patients.

An interesting finding that warrants explanation is that rest increased the pain experience in 13% of patients (Table 5). We assume this may be because a lack of activity allows patients to focus more on their pain.

Research indicates that the assessment and treatment of chronic pain in HD patients are often inadequate [3,30], as nephrologists may not be trained to identify and manage pain effectively [3,11]. A study by Davison involving patients on HD who experience pain revealed that 35% were not receiving any analgesics. Furthermore, only 6% reported effective pain management [26].

In our study, consistent with previous research, 36% of patients did not take medication for their pain. Drug treatment successfully eliminated pain in only 29% of patients.

In contrast to previous research [23,31,32,33,34,35,36,37], our study found that patients on HD did not receive any non-pharmacological treatment or effective adjuvant medications for neuropathic pain, nor did they receive strong opioids for severe nociceptive pain that was resistant to treatment.

Preliminary studies suggest that therapies targeting pain in the dialyzed population could improve their QoL [30,38]. Non-pharmacological analgesic therapies, such as massage, yoga, heat therapy, posture correction, music therapy, acupuncture, breathing exercises, and spiritual coping, can be effective either on their own or in combination with pharmacological treatments [31,32,33,34,35]. In patients undergoing HD, complex pain syndromes require a comprehensive analgesic approach that includes a combination of non-opioid medications, opioids, and adjuvant therapies [29]. According to the World Health Organization (WHO) analgesic ladder [15], non-opioid medications such as metamizole sodium, paracetamol, and non-steroidal anti-inflammatory drugs (NSAIDs) should be the primary choice for drug treatments [36]. Opioids, whether weak or strong, should only be used in dialysis patients for moderate-to-severe pain that cannot be effectively managed with non-opioid analgesics [37]. Non-opioid and opioid analgesics effectively treat nociceptive pain, and a poor response to paracetamol, NSAIDs, and most opioids characterizes neuropathic pain. According to treatment guidelines for neuropathic pain, the initial approach should involve adjuvant medications [1].

Numerous studies highlight the importance of integrated PC for patients undergoing HD. They emphasize a holistic approach to PC, which recognizes that “total pain” includes physical, psychosocial, and spiritual components. Accurate assessments are crucial for effective pain management, as pain is often underdiagnosed and inadequately treated [39], as demonstrated by our study. Comprehensive patient histories, thorough examinations, and assessment tools are essential in PC pain management [25]. PC facilitates the multidisciplinary use of non-pharmacological methods, which can help reduce the side effects associated with drug treatments in frail patients with advanced kidney disease [23], as would have been necessary for our patients. In cases of severe and treatment-resistant pain, such as intractable neuropathic pain, a palliative consultation is essential to determine the appropriate medications, as demonstrated by the complete absence of adjuvant agents and strong opioids in our study. For all patients with serious illnesses, including those with advanced kidney disease, it is essential to carefully consider the potential risks and benefits of therapy in the context of each individual’s situation. Close monitoring for adverse effects and careful dose adjustments are standard practices in specialty PC [3], but despite its importance, research shows that its regular availability for patients with ESKD is severely limited in most countries, including Hungary [7,9,14,40].

## 5. Future Prospects

Our future plans involve enhancing standard nephrological treatment with PC for severe, intractable pain, focusing on previously inadequately treated neuropathic pain and incorporating non-pharmacological methods.

Using these findings, we plan to conduct a multicenter pain assessment across multiple dialysis centers in Hungary to advocate for making national palliative guideline for HD patients.

## 6. Limitations

This study uses the PQRST approach for pain evaluation. While this is a useful clinical tool, it is not a validated, renal-specific research instrument such as the BPI, which would allow greater comparability with other studies.

This study also uses a cross-sectional design and was conducted at a single center, which limits causal inference and generalizability.

Furthermore, the absence of non-pharmacological or adjuvant therapies affects comparability.

## 7. Conclusions

Pain is a common issue for patients undergoing HD, and the level of pain they experience is comparable to that faced by patients with advanced cancer who may receive PC. In our investigation of factors influencing this symptom burden, we found a significant difference in pain intensity between individuals with low and high levels of PTH and CRP. Additionally, we observed a strong correlation between pain intensity and factors such as DM, HT, BMI, and PTH levels. PC is essential for relieving symptoms in patients through comprehensive pain assessments and a multidisciplinary treatment approach that also incorporates non-pharmacological methods with fewer side effects. Pain management should be initiated by nephrologists, as their training in PC is crucial. However, in cases of severe and treatment-resistant pain, such as intractable neuropathic pain, palliative consultations are essential. Our findings highlight the necessity for patients with ESKD to have access to PC.

## Figures and Tables

**Table 1:** The baseline clinical data and pain presence.

*n*	159
Age (years)	65 (±12)
Sex (men)	80 (50%)
Pain	
Yes	91 (57%)
No	68 (43%)

**Table 2:** Analysis of pain severity in different areas experienced by 91 patients.

Severity of Pain (in Several Different Regions)	
Mild (NRS 1–3)	15 (16%)
Moderate (NRS 4–6)	39 (43%)
Severe (NRS 7–10)	41 (45%)

NRS: Numeric Rating Scale.

**Table 3:** Multivariate analyses of clinical data and laboratory results associated with pain intensity.

Parameter	OR	CI 95%	*p*
BMI	2.296	1.019–2.981	0.012
HT	7.932	1.207–14.658	<0.001
DM	7.375	1.917–13.833	0.013
PTH	1.578	1.089–2.236	0.027

BMI: body mass index; HT: hypertension; DM: diabetes mellitus; PTH: parathyroid hormone.

**Table 4:** Location and characteristics of the pain.

**Location**	
Hip/lower limb	50 (55%)
Back	20 (22%)
Shoulder/arm	20 (22%)
Head/neck	18 (20%)
Distal foot	16 (18%)
Waist	15 (16%)
Gluteal region	10 (11%)
Abdomen	9 (10%)
Distal hand	7 (8%)
Chest	6 (7%)
**Characteristics**	
Sharp	28 (31%)
Cramping	22 (24%)
Aching	22 (24%)
Dull	16 (18%)
Numb	15 (16%)
Stabbing	12 (13%)
Throbbing	8 (9%)
Burning	6 (7%)

**Table 5:** Aggravating and relieving factors and the negative effects of pain.

**Aggravating factors**	
Physical activity	46 (51%)
Rest	12 (13%)
HD	3 (3%)
Weather change	3 (3%)
Meal	2 (2%)
**Relieving factors**	
Rest	33 (36%)
Physical activity	16 (18%)
**Negative impact on**	
Physical activity	57 (63%)
Sleep	44 (48%)
Appetite	14 (15%)
Emotion	13 (14%)
Attention	10 (11%)
Relationship	5 (5%)

HD: hemodialysis.

**Table 6:** The medications used for pain management and their effectiveness.

Non-Pharmacological Treatment	0 (0%)
Pharmacological treatment (sometimes in combination)	58 (64%)
NSAIDs	31 (53%)
Metamizole	22 (38%)
Paracetamol	3 (5%)
Weak opioid (tramadol)	13 (22%)
Strong opioid	0 (0%)
Adjuvant treatment	0 (0%)
**Effectiveness of treatment**	
No	1 (2%)
Pain relief	40 (69%)
Pain elimination	17 (29%)

NSAID: non-steroidal anti-inflammatory drug.

## Data Availability

Data are contained within the article.

## Abbreviations

BMI: body mass index; BPI: Brief Pain Inventory; CKD: chronic kidney disease; CRP: C-reactive protein; DM: diabetes mellitus; ESAS-r: Renal: Edmonton Symptom Assessment System Revised Renal; ESKD: end-stage kidney disease; HD: hemodialysis; HT: hypertension; IPOS-Renal: Integrated Palliative Care Outcome Scale Renal; KSC: kidney supportive care; NRS: Numeric Rating Scale; NSAID: non-steroidal anti-inflammatory drug; PC: palliative care; PTH: parathyroid hormone; QoL: quality of life; SD: standard deviation; WHO: World Health Organization.
